# (In)justice in academia: procedural fairness, students’ academic identification, and perceived legitimacy of university authorities

**DOI:** 10.1007/s10734-022-00907-8

**Published:** 2022-08-08

**Authors:** Michał Główczewski, Stanisław Burdziej

**Affiliations:** 1 Institute of Psychology, Nicolaus Copernicus University, Jurija Gagarina 39, Toruń, 87-100 Poland; 2 Institute of Sociology, Nicolaus Copernicus University, Fosa Staromiejska 1a, Toruń, 87-100 Poland

**Keywords:** Procedural fairness, Legitimacy, Trust, Academic identification, Students, Grading

## Abstract

A considerable body of literature has documented the significance of fair treatment in terms of generating trust towards decision-makers across different institutional contexts. It has also been demonstrated that even young children are sensitive to procedural justice, and that experiences of both fairness and unfairness help shape young people’s wider attitudes towards authority. In this paper, we seek to extend these findings into the academic context. We use data from two separate studies of university students in Poland. In study 1 (*N* = 315), using a survey to capture students’ actual experiences, we find that fair treatment was a stronger predictor of perceived legitimacy of university authorities than were fair outcomes. In study 2 (*N* = 751), also using a survey of a nationally representative sample of university students, we demonstrate that this procedural effect is mediated by students’ identification with their university, and that trust in academic authorities translates into higher levels of engagement and lower levels of burnout. Academic identification fully mediated the relationship between both procedural and distributive fairness and engagement and partly mediated the relationship between the two dimensions of fairness and burnout. We conclude that the experience of procedural fairness leads students to more strongly identify with their university and thereby enhances their trust in university authorities.

**Supplementary Information:**

The online version contains supplementary material available at 10.1007/s10734-022-00907-8.

## Introduction

In modern societies, universities function as the key gatekeepers to the attainment of high social status. Grades, degrees, scholarships, and other such rewards increasingly shape individuals’ life chances (Waldow, 2014).^1^ Also, trust in academia impacts trust in science more generally. Thus, fairness and legitimacy of grading and other decisions made in the academic environment are of crucial importance not only to scholars and students but also to the wider society. Therefore, scholars across several disciplines are paying more and more attention to fairness. Research has established that not only fair outcomes (i.e., distributive fairness) but also fair treatment (i.e., procedural fairness) matter greatly to the recipients of allocation decisions in a variety of settings, including citizen-police encounters (Jonathan-Zamir et al., 2015), court proceedings (Tyler, 1984), taxation (Murphy, 2005), doctor-patient relations (Mentovich et al., 2014), and employment practices (Cropanzano, 1993). Indeed, fair treatment may matter more than fair outcomes in some institutional contexts because it is indicative of individuals’ status within their respective groups (Tyler & Blader, 2003). The experience of fairness has consistently been shown as important not only for ethical but also for instrumental reasons, contributing to the perceived legitimacy of decision-making authorities, decision acceptance, and voluntary cooperation (Jackson et al., 2012; Tyler, 1990).

Given the crucial impact of universities in terms of socializing the future elites in societies, it is surprising that there are almost no studies testing the salience of procedural fairness in the academic context. We think that interaction with academic authorities and lecturers is a formative learning experience in itself. It, therefore, matters a great deal whether these experiences are conducive to trust and voluntary cooperation or result in cynicism, burnout, and disengagement. For these reasons, this study aims to systematically test the procedural effect in the academic context.

Our research setting — Poland — both enriches and limits our contribution to scholarship on higher education. On the one hand, we report findings from a national context that is relatively neglected in the literature, dominated by studies from the UK and the USA. Since its democratic transition in 1989, Poland has experienced an exponential rise in access to higher education (Kopycka, 2021). The number of students in higher education institutions has increased almost five times, from just over 400,000 in 1990/1991 to almost 2 million in 2005/2006. Private schools have also proliferated, with many offering only poor-quality services due to the “more is better” approach that was motivated politically by the desire to grant equal access to the underprivileged (see Antonowicz & Pinheiro, 2015). However, despite this effort, the intergenerational reproduction of educational disadvantage has persisted (Herbst & Rok, 2014; Kopycka, 2021). At the same time, the strictly hierarchical organizational culture of most higher education institutions has been retained, particularly in certain fields of study, thus reproducing an elitist model that is deeply out of step with the wider global trends. While other sectors of the Polish economy and society have undergone deep democratization, the universities have continued in their old model. However, by the early 2000s, a “demographic tsunami” (Antonowicz & Gorlewski, 2011) and the challenge of Europeanization resulting from the Bologna process (Dakowska, 2015) have undermined this model. The number of young people aged 19–24 decreased to 2.3 million in 2020, including some 1.2 million university students, finally necessitating more attention to the quality of teaching. In recent years, along with the heightened public scrutiny of other previously trusted institutions (such as the Catholic Church), the universities came under fire following cases of mobbing and harassment. Thus, at the time this paper was written, teacher-student relations at the university were a hotly debated issue in Poland (Story, 2021). At the same time, our focus on Poland, a country where higher education is free and widely available, clearly limits the relevance of our findings for those many settings, where obtaining a university degree is costly and difficult.

## State of the art

### Procedural justice in higher education

Over the past three decades, there has been an explosion of research concerning procedural fairness. One strand has focused on law enforcement, the courts, and other institutions of the justice system (Tyler, 1984, 1990), while another has given considerable attention to fairness in organizations (Ambrose & Schminke, 2009; Colquitt, 2001; Colquitt & Zipay, 2015; Folger & Konovsky, 1989; Leventhal, 1980). However, in the past couple of decades, scholars have been increasingly looking at other institutional contexts. While most recent research still focuses on business organizations and law enforcement, some research has addressed the significance of procedural justice in schools. Much of this work has focused on the *distributive* fairness of grading (see Deutsch, 1979). One example of such a study was carried out by Amemiya and colleagues (2020), who found that students’ trust in teachers predicted their behavior in class.

Somewhat surprisingly, however, relatively few studies have focused on the educational context itself. Kravitz et al. (1997) tested a general model based on Leventhal’s (1980) six elements of procedural fairness in higher education. In their paper, they offer a useful catalog of allocation situations in academia: “a) the acceptance to university); b) the assignment of grades to students; c) the allocation of scholarship, assistantship, fellowship, and work-study money to students; d) the allocation of pay, offices, classes, classrooms, research space, and other resources to faculty; and e) the allocation of funds to academic, social, and athletic programs” (p. 700). This list could be extended to include (f) the evaluation of faculty and (g) the participation of both students and faculty in the strategic decision-making processes at their universities. In the cited study, the authors focused on grading and found that the perceived procedural fairness of grade appeal procedures fostered student acceptance of outcomes.

In another of these rare studies, Reisig & Bain (2016) demonstrated that students who perceived university authorities as legitimate were less likely to cheat on exams, concluding that “the explanatory scope of the process-based model extends rule-breaking beyond criminal justice settings” (p. 83). Burger (2017), in turn, examined the effects of the assessment method (essays vs. examinations) and the instruction method (seminars vs. lectures) on student perceptions of the fairness of the assessment process. He found that the assessment method affected the perceived validity of the grading procedures: essays were perceived to grant students more control over the assessment process, while examinations were perceived as more conducive to reliability and neutrality. Bloch et al. (2021) compared scholars’ perceptions of the organizational justice of academic evaluations in Germany and the UK, highlighting the emancipatory potential of these measures in some contexts. Similarly, Waldow (2014) compared the system of national tests after upper secondary schools in Sweden with the evaluation by independent examination boards in England and assessment by teachers in Germany. He found considerable differences in the understanding of fairness in each of the three countries.

A related issue of interest to scholars is the significance of the cultural context. While there is plenty of evidence that the main procedural effects are largely invariant in relation to gender, age, race, financial status, and other socio-demographic variables, there is a lively debate concerning the impact of wider cultural patterns. Tata (2005) compared American and Chinese student perceptions of grading procedures. Using vignettes, she found that culture can influence the perceptions of voice and interpersonal justice. American students placed more weight on voice, i.e., the opportunity to discuss and appeal the grading decision, while Chinese students were more likely to value dignity and respect.

Several other studies have looked into the significance of perceived procedural justice in legal socialization. Tyler & Trinkner (2018) found that fair treatment nurtures trust and rule-following in children. Tyler and collaborators also demonstrated that even small children display sensitivity to fair procedures and that the quality of experience with representatives of the justice system is particularly consequential during adolescence (Granot & Tyler, 2019; Tyler et al., 2014). Shook et al., (2021) have recently demonstrated that young people who felt they were treated fairly by their defense attorneys viewed the police and the courts as more legitimate.

Finally, it is worth noting that universities across the world are increasingly and explicitly embracing the concept of procedural fairness in their communication with students. Students are informed that their schools understand fairness broadly, specifically embracing the key elements of procedural fairness, such as voice, neutrality, and understanding.^2^ While this practical endorsement of procedural fairness is largely common sense, it also contrasts with the scarce research in the academic context.

### Academic/in-group identification

Tyler and colleagues explained the mechanism of procedural justice using the group engagement theory (also called group-value theory) (Tyler, 2000; Tyler & Blader, 2003; Tyler et al., 1996). They argued that fair treatment reflected an individual’s status within the group to which they belonged. They predicted that the procedural effect would be stronger when the group was important for the individual. Studying the procedural effect in the organizational context, Blader & Tyler (2009) emphasized the role of in-group identification in determining peoples’ behavior within work organizations. Further studies have confirmed that one’s perceptions of procedural fairness can be influenced by the extent of one’s group identification (Radburn et al., 2018).

Interesting findings were determined by Leung et al. (2007), who studied the procedural effect in the group-level decision context. They observed that a higher level of in-group identification reduced the importance of procedural justice but increased the importance of a collective decision outcome. Huo and colleagues (1996) demonstrated that when people strongly identified with the superordinate group (e.g., nation), procedural issues were the dominant predictor of their evaluations of interactions with authorities. Finally, in their research concerning university legitimacy, Reisig & Bain, (2016) argued that in the future, researchers should consider whether student legitimacy perceptions are linked to other outcomes of theoretical interest. As one of these outcomes, they proposed “university identification,” which they operationalized as, for example, being proud of the university one attends or treating it as a personal compliment when someone acknowledges the achievements of one’s university.

### Burnout and engagement in university students

Navarro-Abal et al. (2018) investigated the relations between organizational justice and academic engagement and burnout among Spanish students. They observed that college students who were treated fairly felt more engaged with their studies as well as less emotionally exhausted and less cynical about their studies and the university.

Academic burnout can be defined as the psychological state that develops in relation to educational institutions, and it may affect either teachers or students at any educational level. As in the organizational literature concerning burnout at work, it has been observed that students who began their studies with enthusiasm subsequently came to express a sense of disappointment, lack of energy, feeling of emptiness or failure, low self-efficacy, problems with concentration, and a higher level of cynicism about the further study (Schaufeli et al., 2002). Schaufeli et al. (2002) distinguished three elements of academic burnout: high *emotional exhaustion* — feeling exhausted from one’s studies; high *academic cynicism* — the belief that one’s studies have lost their meaning and thus distancing from studies and university; and low *academic efficacy* — feeling a lack of self-efficacy during one’s studies. Previous research has demonstrated that the sense of procedural and distributive fairness is associated with a lower level of burnout (van Dierendonck et al., 2001) and psychological strain (Francis & Barling, 2005).

Academic engagement, in turn, can be defined as the willingness to participate in student activities, follow the lecturers’ instructions, and study with eagerness (Schaufeli et al., 2002). In the cited study, Schaufeli et al. distinguished three elements of academic engagement: *vigor* — the belief that studying gives one mental strength and emotional vigor; *dedication* — being proud of and enthusiastic towards one’s studies, which is the opposite of academic cynicism; and *absorption* — the state wherein students are absorbed during the study. *Engaged students* usually have more positive attitudes towards learning and taking part in class activities, are more enthusiastic and curious, and take more advantage of the learning opportunities offered (Vollet et al., 2017).

## Present studies

Building on these studies, we hypothesize that procedural justice will significantly shape students’ trust in academia, as well as their behaviors in academic settings. In line with a growing body of research, we expect that students will appreciate voice, neutrality, respect, and understanding even more strongly than they do favorable outcomes (e.g., grades or decisions). We also predict that the students’ experience of procedural justice will be a negative predictor of their sense of academic burnout and a positive predictor of their academic engagement.

We want to investigate whether Navarro-Abal et al. (2018) findings replicate in the context of Polish universities and to extend these observations by proposing a theoretical model, which will explain the mechanisms that account for the positive association between the students’ experience of procedural justice and academic burnout and engagement.

Therefore, we predicted that:

- Hypothesis 1: The students’ experience of procedural fairness would be a stronger predictor of their perceived legitimacy of university authorities than the experience of distributive fairness.
- Hypothesis 2: The relationship between the experience of procedural fairness and the perceived legitimacy of university authorities would be mediated by students’ in-group identification.
- Hypothesis 3a: The experience of procedural fairness would positively predict academic engagement.
- Hypothesis 3b: The experience of procedural fairness would negatively predict academic burnout.

Our main research objective was to see how significant is procedural fairness in the academic setting. We wanted to find out how sensitive are students to fair treatment, given that the favorability of decisions taken by university authorities may be considerable? Our first study was designed to capture the real experience of students in Poland and see whether the procedural effect previously found in many other institutional contexts can also be seen in academia. Our second study was carried out to better understand the mechanisms behind procedural fairness: how the experience of fairness correlates with students’ identification with their university, and with their engagement in their studies? The key theoretical contribution of our paper is to bring key insights from research on people’s experience of fairness to the study of higher education. We do this by proposing and validating a model which explains how procedural and distributive fairness impacts students’ academic engagement and identification, and how fair treatment is conducive to trust in university authorities. Our findings attest to the significance of procedural fairness in the academic context and point to the need for further studies exploring links between fair treatment and student satisfaction, as well as with a host of other desired outcomes.

To verify our hypotheses, we decided to use a cross-sectional design in both studies. Research procedures were reviewed and approved by the relevant ethics committee.^3^ Both studies were conducted in accordance with the American Psychological Association’s (APA) ethics principles and data protection standards.^4^

## Study 1

The aim of study 1 was to investigate the effects of procedural and distributive fairness on the perceived legitimacy of academia using a survey. We hypothesized that the students’ actual experience of procedural fairness would be a stronger predictor of their perceived legitimacy of university authorities than their experience of distributive fairness (H1).

### Method

#### Participants and design

The a priori power analysis using the average social psychology effect size (*r* = 0.21; Richard et al., 2003) determined that a sample size of 173 would achieve a power of 0.80. Three hundred and fifteen participants, 249 women and 66 men, aged 19–44 (*M* = 23.05; *SD* = 3.20), took part in the study. They were students from Polish public universities, recruited via the Ariadna — Polish online research platform, which has been widely used in academic studies (e.g., Górska et al., 2019). The participants answered questions related to their actual experience of procedural and distributive fairness in the university setting and then of their perceived legitimacy of university authorities, as well as questions related to demographics (age, gender, place of living, year of studies).

#### Measures

*Experience of procedural fairness* was measured by own scale consisting of 11 items (e.g., “How often did the lecturers generally provide students with the opportunity to speak in class?”; “During the last academic year, have the lecturers treated the students with respect?”; and “During the last academic year, did the teachers assess students based on their preferences, stereotypes, and prejudices?”) on a response scale from *hardly ever* (1) to *almost always* (7) or *definitely not* (1) to *definitely yes* (7), *α* = 0.88.

*Experience of distributive fairness* was measured by own scale consisting of three items (e.g., “How often have you felt that the lecturer judged you unfairly?”; “How often have you been satisfied with your grades?”; and “How often did you feel that the grades you received during your studies reflected correctly your level of knowledge and skills?”) on a response scale from *hardly ever* (1) to *almost always* (7), *α* = 0.58.

*Perceived university legitimacy* was measured by own scale consisting of four items (e.g., “Most academic lecturers have extensive knowledge”; “The decisions of the university authorities that concerned me were right”; “Academic teachers should always be respected, even if we disagree with them”; and “Academic lecturers deserve respect”) on a response scale from *definitely not* (1) to *definitely yes* (7), *α* = 0.73.

### Results and discussion

The descriptive statistics and correlations are presented in Table 1. We observed a strong, positive, and statistically significant correlation between the experience of procedural fairness and the experience of distributive fairness, and between the experience of procedural fairness and perceived university legitimacy. Additionally, we observed a moderate, positive, and statistically significant correlation between the experience of distributive fairness and perceived university legitimacy.

**Table 1:** Means, standard deviations, and bootstrapped zero-order correlations with 95% standardized confidence intervals (Study 1)

Variables	1	2	3
1. Experience of procedural fairness	-		
2. Experience of distributive fairness	0.59^***^ [0.51, 0.65]	-	
3. Perceived university legitimacy	0.62^***^ [0.55, 0.68]	0.42^***^ [0.33, 0.52]	-
*M*	5.22	4.66	5.27
*SD*	0.83	1.00	0.86

^***^p < 0.001

We tested our hypothesis with a linear regression model (Table 2). We examined students’ experience of procedural and distributive fairness as predictors of their perceived university legitimacy. Perceived university legitimacy was positively predicted by the experience of procedural fairness but not by distributive fairness. This pattern of results remained similar when we controlled for participants’ demographics and their year of studies.

**Table 2:** Bootstrapped regression models with perceived university legitimacy as the dependent variable (Study 1)

Variables	Model 1			Model 2		
	*B* [95% *CI*]	*β*	*p*	*B* [95% *CI*]	*β*	*p*
Experience of procedural fairness	0.58 [0.48, 0.70]	0.56	< 0.001	0.57 [0.47, 0.68]	0.55	< 0.001
Experience of distributive fairness	0.08 [− 0.02, 0.17]	0.09	0.100	0.09 [− 0.01, 0.19]	0.11	0.073
Year of studies				0.01 [− 0.07, 0.09]	0.01	0.796
Age				− 0.02 [− 0.05, 0.01]	− 0.08	0.200
Gender				0.02 [− 0.16, 0.22]	0.01	0.818
*F*	*F*(2, 312) = 97.27, *p* < 0.001			*F*(5, 309) = 39.36, *p* < 0.001		
*R*^2^_adj_	0.38			0.38		

Study 1 offered support for our hypothesis that students’ experience of procedural fairness would be a stronger predictor of the students’ perceived university legitimacy than their experience of distributive fairness. This indicates that the way students are treated by lecturers and university authorities, including ensuring their right to speak and treating them with respect, as well as ensuring impartial decision-making and the clarity and transparency of rules, has a more significant impact on building students’ positive attitudes towards the university and trust in the university authorities than does the favorability of the decisions made on their behalf.

## Study 2

Although study 1 confirmed our key hypotheses, it did not explain what mechanisms account for the positive association between the experience of procedural fairness and students’ perceived university legitimacy. Thus, study 2 was designed to meet three objectives. First, we wanted to replicate the effect we observed in study 1 and verify our Hypothesis 1 on a representative sample of Polish students. Second, we wanted to test academic identification as a mediator of the relationship between the experience of procedural fairness and perceived university legitimacy (H2). Third, study 2 was intended to investigate the effects of procedural and distributive fairness on two forms of attitudes towards the university: academic engagement and academic burnout. We predicted that the experience of procedural fairness would positively predict academic engagement (H3a) and negatively predict academic burnout (H3b).

Moreover, we designed this study during the COVID-19 pandemic, which has caused a global transition to remote teaching and study. Recent studies have documented a higher level of depression and anxiety among university students due to the pandemic and its resulting policies (such as lockdowns; see Aristovnik et al., 2020; Rudenstine et al., 2021). Using data from 62 countries, Aristovnik and colleagues (2020) found that students were generally satisfied with the support provided remotely by the lecturers and staff but still experienced boredom, anxiety, and frustration. The students were also concerned about the effects of the pandemic and remote learning on their future professional careers. Therefore, we decided to control for the fear of COVID-19 as a factor potentially affecting students’ perceptions of fairness in their interactions with teachers.

### Method

#### Participants and design

Seven hundred and fifty-one participants, 445 women and 306 men, aged 18–49 (*M* = 23.45; *SD* = 2.61), took part in the study. They were students from Polish public universities, recruited via the Pollster Research Institute, a Polish online research platform. Participants filled out measures of their experience of procedural and distributive justice, identification with the academic community, perceived university legitimacy, academic burnout (exhaustion, cynicism, academic efficacy), academic engagement (vigor, dedication, absorption), fear of COVID-19, and demographics (age, gender, place of living, year of studies).

#### Measures

*Procedural fairness* was measured by the same scale as used in study 1, *α* = 0.85.

*Distributive fairness* was measured by own scale consisting of seven items (e.g., “How often have you felt that the lecturer judged you unfairly?”; “How often have you been satisfied with your grades?”; or “How often did you feel that the grades you received during your studies reflected correctly your level of knowledge and skills?”) on a response scale from *hardly ever* (1) to *almost always* (7), *α* = 0.80.

*Academic identification* was measured with five items based on Cameron’s Social Identity Scale (Cameron, 2004), e.g., “I have a lot in common with other members of my academic community” or “Overall, being a member of my academic community is an important part of who I am.” The participants indicated to what extent they agreed with the given statements on a scale from *definitely not* (1) to *definitely yes* (7), *α* = 0.86.

*University legitimacy* was measured by own scale consisting of nine items (e.g., “Most academic lecturers have extensive knowledge”; “I trust the authorities of my university”; and “Universities generally serve society well” or “Academic lecturers deserve respect”) on a response scale from *definitely not* (1) to *definitely yes* (7), *α* = 0.92.

*Academic burnout* was measured by the Maslach Burnout Inventory — MBI-GS (Schaufeli et al., 1996), translated for this study. The scale consists of three factors: exhaustion, cynicism, and academic efficacy. Exhaustion was measured by five items (e.g., “I feel emotionally drained by my studies” or “I feel burned out from my studies”) on a response scale from *hardly ever* (1) to *almost always* (7), *α* = 0.86. Cynicism was measured by four items (e.g., “I have become less interested in my studies since my enrollment at the university” or “I doubt the significance of my studies”) on a response scale from *hardly ever* (1) to *almost always* (7), *α* = 0.84. Academic efficacy was measured by six items (e.g., “I can effectively solve the problems that arise in my studies” or “During class, I feel confident that I am effective in getting things done”) on a response scale from *hardly ever* (1) to *almost always* (7), *α* = 0.82.

*Academic engagement* was measured by the Utrecht Work Engagement Scale for Students — UWES-S (Schaufeli et al., 2002), which was translated for this study. The scale consists of three factors: vigor, dedication, and absorption. Vigor was measured by five items (e.g., “When I’m studying, I feel mentally strong” or “When studying I feel strong and vigorous”) on a response scale from *definitely not* (1) to *definitely yes* (7), *α* = 0.88. Dedication was measured by five original items (e.g., “I find my studies to be full of meaning and purpose” or “My studies inspire me”) and three items included for this study (“I am proud of my university”; “I would recommend my friends to study at my university”; and “I highly appreciate the prestige of my university”) on a response scale from *definitely not* (1) to *definitely yes* (7), *α* = 0.90. Absorption was measured by four items (e.g., “Time flies when I’m studying” or “I feel happy when I am studying intensively”) on a response scale from *definitely not* (1) to *definitely yes* (7), *α* = 0.79.

*Fear of COVID-19* was measured by the Fear of COVID-19 Scale (Ahorsu et al., 2020), which was translated for this study. The scale consists of seven items (e.g., “I am most afraid of coronavirus-19” or “I am afraid of losing my life because of coronavirus-19”). The participants indicated to what extent they agreed on the given statements on a scale from *definitely not* (1) to *definitely yes* (7), *α* = 0.88.

### Results and discussion

The descriptive statistics and correlations are presented in Table 3. All our key variables were correlated in the expected directions: both the experience of procedural and distributive fairness were positively correlated with academic efficacy, vigor, dedication, and absorption, and negatively correlated with exhaustion and cynicism. Academic identification was positively correlated with procedural and distributive fairness, university legitimacy, academic efficacy, and all the dimensions of academic engagement. Also, we observed negative correlations between academic identification and the two dimensions of academic burnout—exhaustion and cynicism. University legitimacy was positively correlated with procedural and distributive fairness, academic efficacy, and all the dimensions of academic engagement. Moreover, we observed negative correlations between university legitimacy and exhaustion, and cynicism. The fear of COVID-19 correlated positively with academic identification, vigor, dedication, and absorption, and negatively with procedural and distributive fairness.

**Table 3:** Means, standard deviations, and bootstrapped zero-order correlations (Study 2)

Variables	*M*	*SD*	1	2	3	4	5	6	7	8	9	10
1. Procedural fairness	5.26	0.90	-									
2. Distributive fairness	4.88	0.96	0.68^***^	-								
3. Academic identification	4.47	1.27	0.36^***^	0.36^***^	-							
4. University legitimacy	5.07	1.08	0.71^***^	0.59^***^	0.48^***^	-						
5. Exhaustion	4.34	1.38	− 0.38^***^	− 0.40^***^	− 0.37^***^	− 0.39^***^	-					
6. Cynicism	3.94	1.47	− 0.36^***^	− 0.35^***^	− 0.40^***^	− 0.40^***^	0.65^***^	-				
7. Academic efficacy	4.83	0.99	0.41^***^	0.43^***^	0.49^***^	0.50^***^	− 0.41^***^	− 0.46^***^	-			
8. Vigor	3.42	1.34	0.22^***^	0.28^***^	0.45^***^	0.36^***^	− 0.49^***^	− 0.45^***^	0.58^***^	-		
9. Dedication	4.42	1.24	0.43^***^	0.38^***^	0.59^***^	0.61^***^	− 0.46^***^	− 0.63^***^	0.64^***^	0.62^***^	-	
10. Absorption	4.06	1.32	0.27^***^	0.24^***^	0.43^***^	0.37^***^	− 0.40^***^	− 0.43^***^	0.62^***^	0.83^***^	0.65^***^	-
11. Fear of COVID-19	2.31	1.22	− 0.15^***^	− 0.11^**^	0.09^*^	− 0.04	0.06	0.02	0.01	0.15^***^	0.08^*^	0.12^***^

^*^p < 0.05. ^**^p < 0.01. ^***^p < 0.001

In a further analysis, we took into account all the dimensions of academic engagement and academic burnout as underlying variables. As Schaufeli and colleagues (2002) pointed out, academic engagement should be considered as a second-order variable formed by three dimensions—vigor, dedication, and absorption. Also, Schaufeli and colleagues (1996) indicated that academic burnout was a three-factor construct, consisting of exhaustion, cynicism, and self-efficacy.

To test for mediation effects, we conducted our analyses using Hayes Process Macro for SPSS. Initially, four models were estimated. Model 1 was similar to that tested in study 1, with procedural and distributive fairness as predictors of perceived university legitimacy. Model 2 was intended to verify Hypothesis 2, which assumed that the positive relationship between the experience of procedural justice and perceived university legitimacy would be at least partially mediated by greater in-group identification—i.e., identification with the academic community of one’s university. The objective of model 3 was to verify Hypothesis 3a, which was that the experience of procedural fairness would positively predict academic engagement. Model 4 was intended to investigate Hypothesis 3b, which was that the experience of procedural fairness would negatively predict academic burnout.

In every model, we controlled for participants’ demographics, year of studies, and fear of COVID-19. We conducted our analyses with the use of bias-corrected bootstrapping (with 5,000 re-samples).

Model 1 revealed that perceived university legitimacy was positively predicted by the experience of procedural fairness (*B* = 0.70, *SE* = 0.04, *β* = 0.58, *p* < 0.001) and the experience of distributive fairness (*B* = 0.24, *SE* = 0.04, *β* = 0.21, *p* < 0.001). Thus, we obtained further support for Hypothesis 1. Taken together, these two experiences of justice in academia, along with the control variables, explained 53% of the variability in the perceived legitimacy of university authorities.

In model 2, when we added in-group identification as a mediator, both the positive effect of the experience of procedural fairness (*B* = 0.63, *SE* = 0.04, *β* = 0.53, *p* < 0.001) and the experience of distributive fairness (*B* = 0.18, *SE* = 0.04, *β* = 0.16, *p* < 0.001) on perceived university legitimacy decreased, but remained significant. At the same time, in-group identification served as a positive predictor of perceived university legitimacy (*B* = 0.20, *SE* = 0.03, *β* = 0.23, *p* < 0.001) and was positively predicted by the experience of procedural fairness (*B* = 0.32, *SE* = 0.07, *β* = 0.23, *p* < 0.001) and by the experience of distributive fairness (*B* = 0.30, *SE* = 0.07, *β* = 0.23, *p* < 0.001). In line with Hypothesis 2, the indirect effect of procedural fairness on perceived university legitimacy was positive and significant (*IE* = 0.05, *SE* = 0.01, 95% *CI* [0.03, 0.08]). Interestingly, the positive effect of distributive justice was also mediated by in-group identification (*IE* = 0.05, *SE* = 0.01, 95% *CI* [0.03, 0.08]). In model 2, 17% and 58% of the variability in in-group identification and perceived university legitimacy was explained, respectively.

In model 3, both procedural fairness (*B* = 0.32, *SE* = 0.06, *β* = 0.25, *p* < 0.001) and distributive fairness (*B* = 0.25, *SE* = 0.06, *β* = 0.21, *p* < 0.001) positively predicted academic engagement. Thus, we obtained support for Hypothesis 3a. Taken together, these two experiences with fairness in academia, with the control variables, explained 19% of the variability in academic engagement.

In model 4, both the experience of procedural fairness (*B* =  − 0.30, *SE* = 0.05, *β* =  − 0.27, *p* < 0.001) and the experience of distributive fairness (*B* =  − 0.32, *SE* = 0.05, *β* =  − 0.30, *p* < 0.001) negatively predicted academic burnout. Thus, we obtained support for Hypothesis 3b. Taken together, these two forms of justice experience in academia, with the control variables, explained 27% of the variability in academic burnout.

These results confirmed that the experience of procedural fairness was associated with the perception of university authorities as more legitimate. Those treated fairly more strongly identified with their university had more trust in academic authorities and were more likely to accept their grades and other decisions affecting them. These findings led us to propose two additional hypotheses aimed at illuminating the link between fair treatment, academic identification, university legitimacy, students’ academic engagement, and burnout:

- Hypothesis 4a: The positive effect of procedural fairness on academic engagement would be serially mediated by high academic identification and high perceived university legitimacy.
- Hypothesis 4b: The negative effect of procedural and distributive fairness on academic burnout would be serially mediated by high academic identification and high perceived university legitimacy.

To test for the hypothesized serial mediations, we estimated two additional models. Model 5 was intended to verify Hypothesis 4a, and model 6 was intended to verify Hypothesis 4b.

**Fig. 1:**
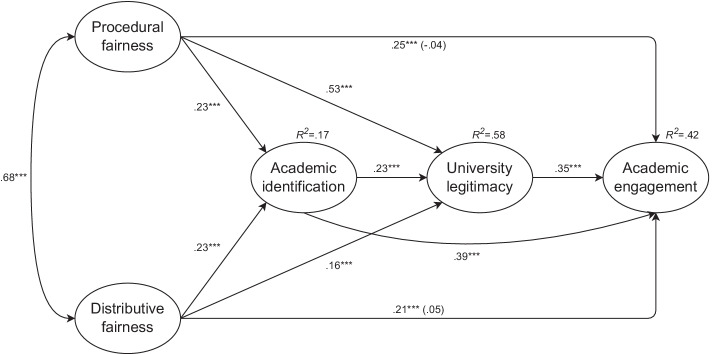
Indirect effect of procedural and distributive fairness on academic engagement by academic identification and university legitimacy (Study 2). Note. Entries are standardized coefficients. ^***^p < 0.001

In model 5 (Fig. 1), we tested procedural and distributive fairness as predictors, in-group identification as mediator 1, perceived university legitimacy as mediator 2, and academic engagement as the dependent variable. Both procedural (*B* =  − 0.05, *SE* = 0.06, *β* =  − 0.04, *p* = 0.398) and distributive fairness (*B* = 0.05, *SE* = 0.05, *β* = 0.05, *p* = 0.322) were nonsignificant predictors of academic engagement. Academic engagement was positively predicted by in-group identification (*B* = 0.35, *SE* = 0.04, *β* = 0.39, *p* < 0.001) and perceived university legitimacy (*B* = 0.37, *SE* = 0.05, *β* = 0.35, *p* < 0.001). At the same time, perceived university legitimacy was positively predicted by the experience of procedural fairness (*B* = 0.63, *SE* = 0.04, *β* = 0.53, *p* < 0.001), the experience of distributive fairness (*B* = 0.18, *SE* = 0.04, *β* = 0.16, *p* < 0.001), and in-group identification (*B* = 0.20, *SE* = 0.02, *β* = 0.23, *p* < 0.001). In-group identification was positively predicted by the experience of procedural fairness (*B* = 0.32, *SE* = 0.07, *β* = 0.23, *p* < 0.001) and the experience of distributive fairness (*B* = 0.30, *SE* = 0.07, *β* = 0.23, *p* < 0.001).

The serial indirect effect of the experience of procedural justice via in-group identification and perceived university legitimacy on academic engagement was positive and significant (*IE* = 0.02, *SE* = 0.01, 95% *CI* [0.01, 0.03]), which supported Hypothesis 4a. In line with our expectations, the positive relationship between the experience of procedural fairness and academic engagement was mediated by increased in-group identification and increased perceived university legitimacy. The two single-mediator positive indirect effects of procedural fairness were also significant: procedural fairness was positively associated with academic engagement via both increased in-group identification (*IE* = 0.09, *SE* = 0.02, 95% *CI* [0.05, 0.13]) and increased perceived university legitimacy (*IE* = 0.19, *SE* = 0.03, 95% *CI* [0.13, 0.25]). The positive relationship between distributive fairness and academic engagement was serially mediated by increased in-group identification and increased perceived university legitimacy (*IE* = 0.02, *SE* = 0.01, 95% *CI* [0.01, 0.03]). Simple mediations of this effect via increased in-group identification (*IE* = 0.09, *SE* = 0.02, 95% *CI* [0.05, 0.13]) and increased perceived university legitimacy (*IE* = 0.06, *SE* = 0.01, 95% *CI* [0.03, 0.09]) were also significant. In total, 42% of the variability in academic engagement was explained.

**Fig. 2:**
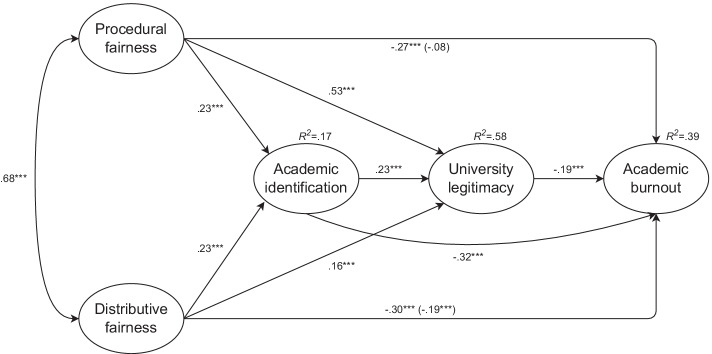
Indirect effect of procedural and distributive fairness on academic burnout by academic identification and university legitimacy (Study 2). Note. Entries are standardized coefficients. ^***^p < 0.001

In model 6 (Fig. 2), we tested the experience of procedural fairness and the experience of distributive fairness as predictors, with in-group identification as mediator 1, perceived university legitimacy as mediator 2, and academic burnout as the dependent variable. The experience of procedural fairness was a nonsignificant predictor of academic burnout (*B* =  − 0.09, *SE* = 0.06, *β* =  − 0.08, *p* = 0.096). Academic burnout was negatively predicted by the experience of distributive fairness (*B* =  − 0.20, *SE* = 0.05, *β* =  − 0.19, *p* < 0.001), in-group identification (*B* =  − 0.26, *SE* = 0.03, *β* =  − 0.32, *p* < 0.001), and perceived university legitimacy (*B* =  − 0.18, *SE* = 0.04, *β* =  − 0.19, *p* < 0.001). The results of the experience of procedural fairness, the experience of distributive fairness, and in-group identification as predictors of perceived university legitimacy and the experience of procedural and distributive fairness as predictors of in-group identification were the same as described in model 5.

The serial indirect effect of the experience of procedural fairness via in-group identification and perceived university legitimacy on academic burnout was negative and significant (*IE* =  − 0.01, *SE* = 0.003, 95% *CI* [− 0.02, − 0.004]), which supported Hypothesis 4b. In line with our expectations, the negative relationship between procedural fairness and academic burnout was mediated by increased in-group identification and increased perceived university legitimacy. The two single-mediator negative indirect effects of procedural fairness were also significant: procedural fairness was negatively associated with academic burnout via both increased in-group identification (*IE* =  − 0.07, *SE* = 0.02, 95% *CI* [− 0.11, − 0.04]) and increased perceived university legitimacy (*IE* =  − 0.10, *SE* = 0.03, 95% *CI* [− 0.15, − 0.05]). The negative relationship between distributive fairness and academic burnout was serially mediated by increased in-group identification and increased perceived university legitimacy (*IE* =  − 0.01, *SE* = 0.003, 95% *CI* [− 0.02, − 0.004]). Simple mediations of this effect via increased in-group identification (*IE* =  − 0.07, *SE* = 0.02, 95% *CI* [− 0.11, − 0.04]) and increased perceived university legitimacy (*IE* =  − 0.03, *SE* = 0.01, 95% *CI* [− 0.05, − 0.01]) were also significant. In total, 39% of the variability in academic burnout was explained.

## General discussion

The present research aimed to investigate the link between students’ experience of procedural and distributive fairness during their studies and their perceived legitimacy of academia, as well as their academic engagement and academic burnout. Based upon previous studies on the procedural effect in the court system (e.g., Burdziej et al., 2019; Tyler, 1984) and policing (e.g., Hough et al., 2013; Jackson et al., 2012), we hypothesized that the experience of procedural fairness would significantly shape students’ attitudes towards university authorities. We also predicted it would positively correlate with greater students’ identification with their university and academic engagement, as well as negatively correlate with academic burnout. Our results provide evidence in favor of all the above hypotheses. Using two samples of university students, we have demonstrated the salience of fair treatment in the academic context. Students place more value on fair decision-making procedures, such as the opportunity to speak and appeal, be treated in a neutral and respectful way, and so forth, than on distributive fairness, i.e., the grades they receive and other decisions affecting them.

In study 1, we examined the relationship between students’ experience of procedural and distributive fairness and their perceived university legitimacy. We observed that procedural fairness was a stronger predictor of the perceived legitimacy of academic authorities than distributive fairness, which supported Hypothesis 1. These results confirmed the findings from numerous studies examining the link between procedural fairness and the legitimacy of various public institutions (e.g., Mazerolle et al., 2013; Rottman & Tyler, 2014). Moreover, our results support the conclusions of Reisig and Bain (2016), who tested the Tyler-Jackson three-dimensional model of legitimacy in the university context. They argued that university legitimacy can be established through lecturers and university authorities treating students according to the principles of procedural fairness. Tyler (2011) advocated treating the encounters between authorities and subordinates as “teachable moments,” which can strengthen or damage the perceived legitimacy of institutions. Our findings show that students who are treated with respect and offered opportunities to speak perceive academic authorities as more legitimate, are more prepared to accept the grades they receive and other decisions that affect them, and are less likely to complain about these grades or appeal these decisions.

In study 2, we wanted to further investigate what psychological mechanism might account for the relationship between the experience of procedural fairness and the perceived legitimacy of university authorities. We observed that in-group identification, i.e., the identification with the academic community of one’s university, was a positive mediator of that relationship, which supported Hypothesis 2. The experience of being treated fairly was also linked with increased academic identification, and academic identification, in turn, was associated with stronger perceived university legitimacy. These results are in line with the group engagement theory (Tyler & Blader, 2003), which states that fair treatment reflects an individual’s status within the group to which they belong. As Blader and Tyler noted elsewhere (2009), in-group identification also plays a vital role in determining the behavior of employees. Thus, students who are treated fairly tend to see themselves as an important part of their academic community and consider their student status a vital part of their identity. They feel proud of being part of their academic community and view this as important for them. This can lead to the increased perceived legitimacy of university authorities, greater decision acceptance, and greater trust in academia more generally.

Additionally, we examined the relationship between the experience of procedural fairness and two attitudes towards university and university studies: academic engagement and academic burnout. We observed that procedural fairness was a strong and positive predictor of academic engagement, as well as a negative predictor of academic burnout. These results supported Hypotheses 3a and 3b. We, therefore, replicate the findings of Navarro-Abal et al. (2018) in the context of Polish higher education. The experience of being treated fairly can bolster students’ engagement during their studies, including their dedication to academia, greater involvement in lectures and class activities, engagement in various students’ associations and clubs, and commitment to studying. On the other hand, the experience of procedural fairness also decreases their sense of burnout, which includes a low sense of self-efficacy, exhaustion with studies, cynicism, and feeling of a lack of meaning in pursuing further studies.

Finally, we examined the mechanisms that account for the relationship between the experience of procedural fairness and academic engagement and burnout. We demonstrated that this relationship was serially mediated by increased in-group identification and increased university legitimacy, a finding which supported Hypotheses 4a and 4b. Thus, students who are treated with respect and offered opportunities to speak more strongly identify with their academic community, which, in turn, promotes their trust in academic teachers and authorities and leads to greater academic engagement and lower academic burnout. Finally, while it will still need to be empirically demonstrated, we expect that the ultimate consequence of procedural fairness in academia will be increased trust in scientists and science more generally.

The present studies contribute to the existing literature on higher education in several ways. First of all, our studies demonstrate the significance of procedural fairness in the academic context. Kravitz et al. (1997) and Reisig and Bain (2016) indicated that this context has been neglected and constitutes a gap in the procedural justice literature. Secondly, studies concerning procedural fairness were conducted mainly in Western countries. Thaman (1999) pointed out that the concept of procedural justice was rooted in the Anglo-Saxon tradition and developed as a result of the model of the adversarial trial before the grand jury. Brockner et al. (2001) argued that the procedural effect was stronger in prosperous and stable societies with a low power distance orientation. There are only a few works on the procedural effect in post-transformation countries, and they mainly concern the legal context as well, such as the courts (Poland; Burdziej et al., 2019), police (Slovenia; Reisig et al., 2013), and taxation (Poland; Niesiobędzka, 2014). Therefore, another contribution of our research is the location of studies in the Polish context, a country with a relatively recent experience of political transformation. Previous studies have found that students value fair treatment and expect university figures to combine knowledge transmission with care (Anderson et al., 2020). We extend these findings by identifying the underlying mechanism that explains why fair treatment may foster trust and legitimacy, as well as facilitate academic engagement and decrease burnout.

Finally, our study has practical consequences. As universities worldwide struggle to attract the best students and strive for academic excellence, our results highlight the importance of the subjective perceptions of fairness resulting from procedurally just decision-making processes.

### Limitations of the study and future directions

The presented studies are not without limitations. Because of the cross-sectional design and the relative order of our mediators, we cannot draw strong conclusions in terms of causality. The solution to that could be, for instance, a longitudinal study with four points of measurement, which would allow researchers to test serial longitudinal mediation (Selig & Preacher, 2009).

Our findings may be partly explained by our research setting. Crucially, public higher education in Poland is free. This may mean that students are prepared to accept less quality than in those countries where a degree involves a considerable investment. Private universities greatly depend on tuition and thus could be reasonably expected to care more about the quality of teaching. Therefore, subsequent research should compare the experience of students in public and private institutions. Also, the recent overabundance of graduates in Poland has resulted in a clear devaluation of university degrees. Unlike in the 1990s and earlier, a degree no longer guarantees a good job. Employers have learned to vet experience and skills, rather than merely look at grades. Therefore, any academic decisions—around grades, rewards, prizes, etc.—may carry less weight. Previous research on fairness shows that the stakes matter: usually, the higher the stake, the higher the significance of fair outcomes. Thus, further research could systematically look at the subjective perceptions of the importance of the decisions received by students, e.g., by controlling for subject areas (for example, the stakes may be higher for medical students).

In early 2020, the COVID-19 pandemic caused a global transition to remote teaching and study. This particular model of teacher-student interaction may influence students’ perceptions of what is fair. There are early signs that the resulting digitalization of education has led to an “emergency pedagogy” in the sense of the reduced quality of teaching and has exacerbated earlier trends towards the commodification of higher education (Komljenovic, 2020). While we have included the fear of COVID-19 as a possible factor predicting students’ experience of burnout and engagement in study 2 and ensured that our items captured a wide spectrum of student experiences, including remote interactions, it is possible that the particular context of the pandemic has affected our respondents’ experience with university study in ways we need yet to identify.

## Conclusions

Our study shows that students deeply care about fair treatment when evaluating their interactions with teachers and academic authorities. We demonstrate that procedural fairness matters even more strongly than fair outcomes in terms of grades and favorable decisions. These findings will be of particular interest to academic teachers and university managers. In the face of increasing competition for the best student talent (Marginson, 2006) and the best quality of teaching and scholarship, teachers may consider addressing the increasing expectations of students in terms of respectful treatment, greater transparency, and the opportunity to express their views, especially in the process of grading. Various academic bodies will be challenged to design their decision-making processes in such a way as to promote voice, respect, neutrality, and understanding among those who receive these consequential decisions. Furthermore, all will profit from recognizing that university studies, among other factors, teach students how to treat other people, a crucial skill in the contemporary world. Greater focus on the subjective perceptions of the fairness of decision-making will foster trust in academic teachers and authorities, and hopefully, also trust in science and scholarship itself.

Our studies increase our understanding of the significance of students’ experience of justice for their overall satisfaction with university education, as well as other factors such as their engagement and identification. We were able to demonstrate these links in the context of Poland, where higher education is free of charge and widely available. Further research on justice in academia is needed to see whether our findings are replicated in those national contexts, where higher education is costly and beyond the reach of many. There are reasons to expect that in those countries, where prestigious diplomas are career launchpads, students’ tolerance for unfair treatment will be greater. They may perceive any injustice experienced during their education as a necessary transaction cost of getting ahead in their life. Differences may also regard studied disciplines. For example, it is safe to assume that medical or law degrees are perceived as bringing higher rewards than degrees in human and social sciences; therefore, students of law and medicine may be more prepared to tolerate arbitrary and disrespectful treatment. The more desired this resource by students, the more ready they could be to pay a price for getting them. Many elite organizations — including, e.g., military units — display similar characteristics, expecting newcomers to deserve inclusion, sometimes by surviving degrading treatment.

Paradoxically, thus, one could say that the significance of fair treatment for Polish students may negatively attest to the quality of education at Polish universities and the rewards they offer to students. This suggests further research on students’ experience of fairness and its correlates should pay close attention to the area of study and level of academic competition.

## Supplementary Information

Below is the link to the electronic supplementary material.

Supplementary file1 (SAV 21 KB)

Supplementary file2 (SAV 160 KB)

## Data Availability

All data and analyses can be found on the Open Science Framework: https://osf.io/hgmsy/?view_only=0a6c2b2540cf4f248cdc4e306d521487.

## Footnotes

1 Waldow (2014), in his study comparing the perceptions of procedural justice in high school grading in England, Germany, and Sweden, observed that: “In societies following a meritocratic ideal, educational certificates and examinations play a key role in allocating life chances to individuals. Procedures for allocating life chances need to be perceived as fair by those concerned if examinations and certificates are to possess legitimacy.”.

2 See, e.g., the website of Queens University in Canada, https://www.queensu.ca/ombuds/appeal-process/procedural-fairness

3 Ethics Committee of the Faculty of Philosophy and Social Sciences at Nicolaus Copernicus University in Torun, Poland (decision 41/2021).

4 Datasets used in both studies, as well as statistical analyses conducted and models tested, are posted on the website of the Open Science Framework (OSF), https://osf.io/hgmsy/?view_only=0a6c2b2540cf4f248cdc4e306d521487
